# Enhancing Postoperative Outcomes After Metabolic Bariatric Surgery: a Pilot Study of Inhibitory Control Training, Transcranial Direct Current Stimulation and Psychosocial Aftercare

**DOI:** 10.1007/s11695-026-08828-6

**Published:** 2026-07-15

**Authors:** Sarah Alica Rösch, Carsten Thiele, Therese Reinstaller, Tino Zähle, Kathrin Schag, Katrin Giel, Christian Plewnia, Johann Steiner, Florian Junne, Susanne Vogt

**Affiliations:** 1 University Clinic for Psychosomatic Medicine and Psychotherapy, University Medicine, Otto-von-Guericke-University Magdeburg, Medical Faculty, Magdeburg, Germany; 2 Partner Site Halle-Jena-Magdeburg, German Center for Mental Health (DZPG), Magdeburg, Germany; 3 Department of Neurology, University Medicine, Otto-von-Guericke-University Magdeburg, Magdeburg, Germany; 4 Center for Behavioral Brain Sciences (CBBS), Magdeburg, Germany; 5 Institute for Medical Psychology, Otto-von-Guericke University, Magdeburg, Germany; 6 University Clinic for General, Visceral, Vascular and Transplant Surgery, University Hospital, Otto von Guericke University Magdeburg, Magdeburg, Germany; 7 Partner Site Tübingen, German Center for Mental Health (DZPG), Tübingen, Germany; 8 Department of Psychosomatic Medicine und Psychotherapy, Medical University Hospital Tübingen, Eberhard Karl University Tübingen, Tübingen, Germany; 9 Center of Excellence for Eating Disorders (KOMET), Tübingen, Germany; 10 Department of Psychiatry and Psychotherapy, Medical University Hospital Tübingen, Eberhard Karl University Tübingen, Tübingen, Germany; 11 Department of Psychiatry, University Medicine, Otto-von-Guericke-University Magdeburg, Magdeburg, Germany; 12 Laboratory of Translational Psychiatry, University Hospital, Otto-von-Guericke-University Magdeburg, Magdeburg, Germany; 13 Center for Health and Medical Prevention (CHaMP), Magdeburg, Germany

**Keywords:** Transcranial direct current stimulation (tDCS), Metabolic bariatric surgery (MBS), Obesity, Impulsivity, Inhibitory control training, Neuromodulation, Non-invasive brain stimulation (NIBS), Prefrontal cortex, Self-regulation, Weight loss maintenance

## Abstract

**Introduction:**

Heightened food-specific impulsivity, associated with prefrontal cortex hypoactivity, may impair outcomes following metabolic bariatric surgery (MBS). This pilot study examined the feasibility of transcranial direct current stimulation (tDCS) targeting the right dorsolateral prefrontal cortex (dlPFC) combined with an individualized food-specific inhibitory control training addressing behavioral impulsivity alongside psychosocial follow-up (FU) care to support early postoperative lifestyle adaptation.

**Methods:**

Within 18 months post-MBS, *n* = 19 patients were randomized to 6 sessions of verum or sham tDCS over the right dlPFC combined with inhibition training and psychosocial group sessions. Acceptability, feasibility, food-specific impulsivity and diverse secondary outcomes (e.g., quality of life) were assessed 4 weeks (t1) and 3 months (t2) post-tDCS through (BS-specific) interviews, validated questionnaires and blood samplings.

**Result:**

Retention (67 – 80%) and satisfaction (*M*> 4.38, 1 – 5) were high, improvements in eating behavior and impulse control moderate (*M *= 3.56, 1 – 5) and side effects negligible (*M*= 1.46, 1 – 5). Both groups showed moderate improvements in behavioral impulsivity, self-reported perceived hunger and quality of life, body-mass-index and endocrine markers.

**Conclusion:**

Potential effects may have been attenuated through the small, highly motivated, treatment-seeking sample and the rapidly evolving early postoperative phase. Future fully powered trials should include neurobiological outcome measures that may capture tDCS-induced neural changes earlier and extend FU intervals to clarify efficacy and optimize postbariatric stimulation protocols. Incorporating dismantling designs would further help to disentangle the specific contributions of tDCS, the inhibitory control training and psychosocial care.

**Supplementary Information:**

The online version contains supplementary material available at 10.1007/s11695-026-08828-6.

## Key Points

Early post-MBS care integrated psychosocial support, tDCS and inhibition training.

The intervention was feasible, safe, well-accepted, and improved health-related quality of life and eating.

Neurobiological markers may detect neural tDCS effects preceding behavioral effects.

Early postoperative dynamics may limit neuromodulation detectability.

**Supplementary Information:**

The online version contains supplementary material available at 10.1007/s11695-026-08828-6.

## Introduction

While metabolic bariatric surgery (MBS) is increasing in Germany [1] and stabilizing worldwide [2], postoperative follow-up (FU) programs have lagged behind comparatively. Behavioral lifestyle FU interventions are not covered by public health insurance and their effectiveness remains unclear, with evidence limited to benefits for managing uncontrolled and emotional eating [3–6]. In clinical practice, most FU support is provided by surgeons [7] and focuses on weight loss and nutritional deficiencies [8, 9]. International clinical guidelines typically recommend the most intensive FU care within the first postoperative year [9–11], although, adherence to FU care during this period is often suboptimal due to logistical barriers, competing demands, or low perceived need [12]. Moreover, in current clinical practice and research, treatment success continues to be predominantly defined by the body-mass-index (BMI), despite accumulating evidence advocating for a more holistic approach which considers psychosocial outcomes like health-related quality of life (hrQOL) and endocrine markers [13–17]. The one-sided focus on weight loss, lack of tailored FU treatments and a potential mismatch between intervention delivery and patient needs in the early postoperative phase likely limits effective weight loss and improvements in hrQOL and mental health [18–20].

Although partially controversial, systematic reviews [21–23] as well as cross-sectional [24] and longitudinal analyses [25] have linked impulsivity, neuronally reflected in prefrontal cortex (PFC) hypoactivity, along with inhibitory control deficits to less favorable trajectories after MBS, both in terms of weight outcome and hrQOL. While interventional evidence targeting these neurobehavioral peculiarities in MBS remains rare, meta-analyses in related eating and weight disorders demonstrated improved weight loss, craving and binge eating behaviors following non-invasive brain stimulation (NIBS) targeting PFC dysfunction [26–29]. To date, only two pilot studies (*n* = 10–12) applied NIBS prior to MBS. One study administered repetitive transcranial magnet stimulation (TMS) over the left dorsolateral PFC and reportable favorable effects of active versus sham TMS on neural reward processing [30], but not on the relative reinforcing value of food as primary outcome [31, 32]. In another, unpublished study, active versus sham transcranial direct current stimulation (tDCS) improved reaction time in an inhibition task and weight and increased inhibitory control network engagement [33]. Although both studies were conducted preoperatively, evidence identifying impulsivity, inhibitory control deficits, and maladaptive eating behavior as persistent risk factors for poorer outcomes after MBS [24, 25, 34, 35] provides a strong rationale for exploring NIBS in FU care.

Among techniques, tDCS represents a particularly promising approach due to its availability, tolerability, and ease of integration into routine care. Meta-analyses indicated beneficial effects of tDCS on weight-related outcomes, food craving, and eating behaviors in obesity and binge-eating disorder [26, 36, 37], which seemed strongest and more durable when combined with concurrent tasks activating the targeted neural networks [38–43], e.g., prefrontal inhibition processes. In the context of MBS, such a task-enhanced tDCS approach is well aligned with the need for multi-targeted interventions that address not only PFC hypoactivity, but also inhibitory control deficits and psychosocial and nutritional demands [44–46]. As outlined above, postoperative interventions were repeatedly recommended in the first year to prevent weight regain and recurrence of dysfunctional eating from a clinical perspective [3, 12, 47, 48]. Guided by these considerations, we performed the first explorative pilot study examining the feasibility and acceptability of tDCS combined with an inhibitory control training (ICT) and a psychosocial intervention within the first 18 months after MBS, targeting the early postoperative phase. Additive effects on food-specific impulsivity and diverse other outcomes were additionally explored.

## Materials and Methods

### Patients and Procedure

Patients ≥ 18 years with sleeve gastrectomy in the past 18 months (Supplementary Methods) were recruited from the Center for Obesity and Metabolic Surgery of the University Clinic for General, Visceral, Vascular and Transplantation Surgery between July and December 2024. All patients provided written informed consent prior to participation. The double-blind, sham-controlled design included one baseline (t0) and two further assessments 4 weeks (t1) and 3 months after the last tDCS session (t2). Before each assessment, patients were asked to fast overnight for ≥ 10 h. At assessments (Supplementary Table S1), (a) eating disorder psychopathology and mental disorder comorbidity were assessed through objective interviews and validated questionnaires, (b) further outcomes (e.g., food-related cravings) were operationalized through validated questionnaires, (c) weight was objectively measured on a validated scale, and (d) blood samples were drawn before and 30 min after a standardized test meal (125 ml, 300 kcal; 12 g of protein, 12 g of fat, 37 g of carbohydrates, Nutricia Fortimel Compact, Nutricia Milupa GmbH, Hamburg, Germany).

After t0, patients were randomized to six sessions of verum or sham tDCS combined with an ICT twice weekly and monthly psychosocial group sessions delivered by a psychotherapist and qualified dietician ([49] and Supplementary Material for full details).

### tDCS Sessions and Inhibitory Control Training

Before the first tDCS session, patients received a short psychoeducation, explaining the mechanisms of the training, the stimulation, and the transfer of training effects to everyday life. At t0, patients rated their liking of and appetite for *n =* 40 pictures of high-caloric foods [50] on a 5-point Likert scale (1 = *not at all*/*very unappetizing*; 5 = *very much/appetizing*). The 20 pictures with the highest individual ratings were used for the ICT.

Before each tDCS session, 5 × 7 cm-sized electrodes were prepared with a Ten20 conductive paste (Weaver and Company, Aurora, CO, USA) and connected to a battery-driven stimulator (DC-Stimulator Plus, NeuroConn GmbH, Ilmenau, Germany). Rubber electrodes of a unipolar montage were mounted in both groups, with the shorter side of the anode placed horizontally at F4 in accordance with the international 10–20 system [51]. Another same-sized electrode serving as the cathode was positioned extracephalic on the left deltoid muscle.

Following a 5 s fade in, current was applied for 43 s before the ICT in both groups. This mimicked tDCS-specific sensations (e.g., itching) that typically dissipate quickly [52, 53], ensuring comparable experiences for patients in both groups. The “study mode” of the stimulation device enabled operator-blinded activation, guaranteeing successful blinding of the procedure. Study therapists used a 5-digit code from a computer-generated list that automatically triggered the appropriate condition without requiring further input. At t1, patients were asked to guess their assigned condition (i.e., verum vs. sham tDCS) and specify their level of confidence in their guess on a 0–100% scale to assess the success of masking.

**Fig. 1:**
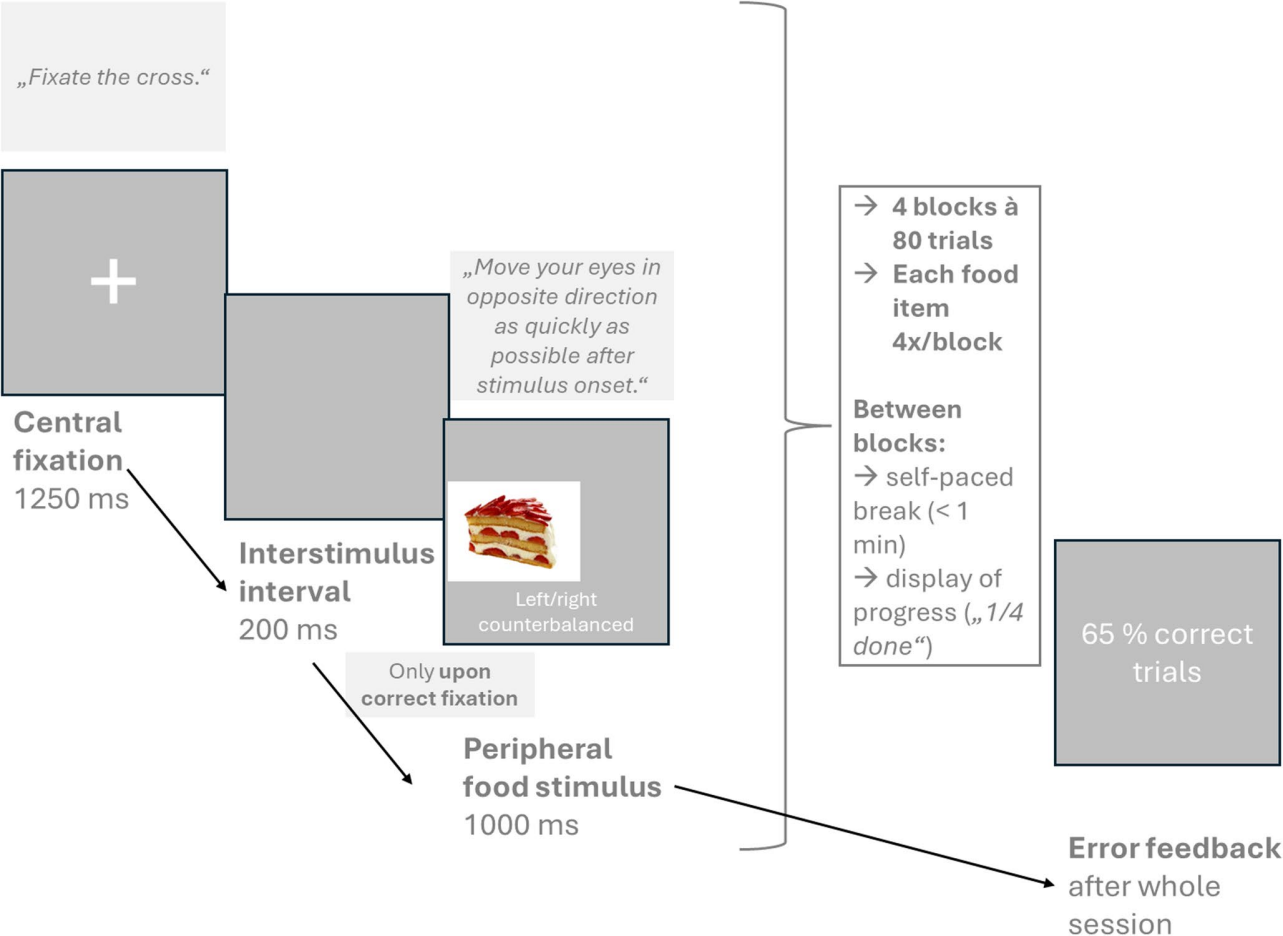
Schematic overview of the inhibitory control training

The ICT [42, 43] began with a central fixation cross displayed for 1250 ms followed by a 200 ms interstimulus interval (Fig. 1). Only if the patient had correctly fixated on the fixation cross, an individual food picture was shown in the peripheral visual field slightly to the left or right of the fixation cross for 1000 ms, with presentation locations counterbalanced between left and right. Patients were asked to perform an antisaccade, i.e., to look in the opposite direction as quickly as possible after presentation of the food stimulus. Each antisaccade away from the food picture was marked as a correct response. Feedback on the percentage of errors was given after each session. Each of the 20 selected food stimuli was presented four times per block across four blocks, resulting in 320 total trials. Patients were allowed to pause for up to 1 min between blocks. Gaze behavior during ICT was tracked through an Eyelink 1000 (SR Research, Ottawa, Ontario, Canada) with a sampling rate of 500 Hz and gaze position accuracy of 0.25–0.50°.

### Outcomes

Primary outcomes encompassed (a) feasibility and acceptability and (b) food-specific impulsivity. The former were depicted in the percentage of included from eligible patients at t0, the drop-out rate through t0 to t2 and a self-developed questionnaire on satisfaction (1 = *not at all satisfied/worsened*, 5 = *very satisfied/clearly improved*) and side effects (1 = *not at all*, 5 = *extremely*). Food-specific impulsivity was operationalized through the error rate and latency in the ICT, the Three Factor Eating Questionnaire (TFEQ; [54, 55]), assessing *restraint of eating behavior*, *disinhibition of control* and *perceived hunger* on 51 items and an own translation of the Food Craving Acceptance and Action Questionnaire (FAAQ; [56], measuring the *ability to regulate eating despite urges and cravings* and the *desire to maintain internal control over eating thoughts* on 10 items.

Secondary outcomes encompassed cravings, hrQOL, eating disorder and general psychopathology, general impulsivity, mental disorder comorbidity, weight, side effects of tDCS and endocrine markers ghrelin and glucagon-like peptide-1 (GLP-1; Supplementary Methods and Supplementary Table S1).

### Statistical Methods

All analyses were conducted in R version 4.4.2. Primary outcomes were analyzed in the intent-to-treat sample using linear mixed-effects models estimated using restricted maximum likelihood with fixed effects of assessment (sessions 1–6 for eye-tracking; t0, t1, t2 for further outcomes), Stimulation (verum, sham), their interaction, and random intercepts for patients to account for within-subject dependencies. Degrees of freedom and statistical inference for fixed effects were obtained using Satterthwaite’s or Kenward-Rogers approximation depending on the model component. For missing data in primary outcomes, multiple imputation was performed at the scale level. Given the pilot nature of the study and the limited sample size, analyses were conducted in an exploratory, non-confirmatory framework, with an emphasis on effect sizes and confidence intervals rather than dichotomous significance testing. Effect sizes were reported as partial eta-squared (η²ₚ) with 95% confidence intervals.

## Results

### Baseline Characteristics

**Fig. 2:**
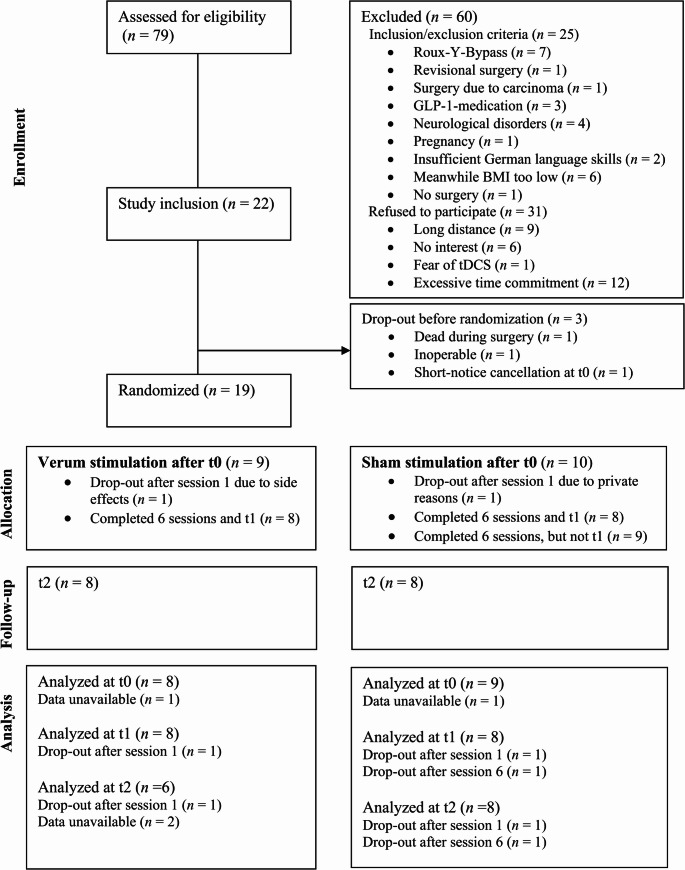
CONSORT patient flow chart

The biologically female, middle-aged sample was enrolled several months after MBS, mostly presented with severe obesity and on average four somatic comorbidities (Supplementary Table S2). Approximately one third had ≥ 12 years of education, one third reported a monthly household income below €1,500, and most patients were part-time or unemployed. Two patients of the sham group reported concomitant psychotherapy unrelated to eating behavior. Patients were highly motivated; weight loss was the primary reason for participation. Three patients (15.79%) dropped out before t1 (Fig. 2).

### Primary Outcome: Feasibility and Acceptability

At t1, retention was high (verum: 88.89% at t1, 66.67% at t2; sham: 80% across assessments; Fig. 1) and both groups provided high ratings for satisfaction (*M* = 4.38, *SD* = 0.50), motivation (*M* = 4.75, *SD* = 0.58), as well as improvements in eating behavior (*M* = 3.56, *SD* = 0.73) and impulse control (*M* = 3.56, *SD* = 0.81), without any differences between groups, *W* < 40, *p* >.349 (Supplementary Results for further items). The most valued elements of the program included group discussions, behavioral strategies, and stimulation, whereas blood samples and meal selection were most often reported as least important. Participation was considered overall meaningful (*M* = 3.94, *SD* = 0.85).

Across groups, 56% of patients thought they had received active stimulation (50% in the verum vs. 62.5% in the sham group, OR = 0.62, *p* = 1), suggesting successful blinding. Technical problems occurred once in the verum group (stimulation device not working in two sessions). Side effects were mild (*M* = 1.46, *SD* = 0.46) and descriptively higher in the sham (*M* = 1.68, *SD* = 0.49) than the verum group (*M* = 1.25, *SD* = 0.34), *r* =.26, *W* = 13.5, *p* =.054. No serious or persistent adverse effects were reported.

### Primary Outcome: Food-Specific Impulsivity

**Fig. 3:**
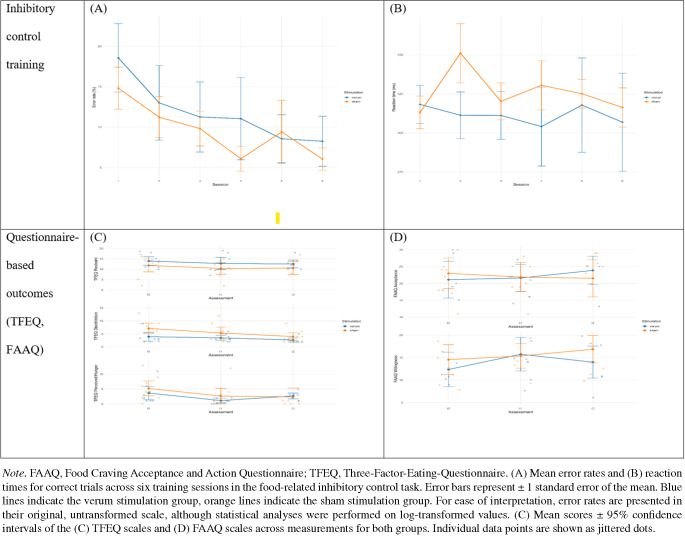
Results for primary outcomes

Preliminary data suggested reduced error rates, *F*(1, 70.52) = 23.52, *p* <.001, η²ₚ = 0.12, 95% CI [0.04, 0.29], and reaction times, *F*(1, 69.73) = 5.80, *p* =.019, η²ₚ = 0.07, 95% CI [0.01, 0.19] across groups over sessions in the ICT and self-reported reduced TFEQ-based hunger from t0 to t1, *t*(27.6) = − 2.31, *p* =.028, η²ₚ = 0.16, 95% CI [0.00, 0.41] (Table 1; Fig. 3).

**Table 1:** Effects of group and assessment on primary outcomes

Outcome	Effect	Test Statistics	*p*	η²ₚ	95% CI η²ₚ ^a^
Inhibitory control task: error rate	Session^b^	*F*(1, 70.52) = 23.52	< 0.001	0.12	[0.04, 0.29]
	Stimulation	*F*(1, 25.87) = 0.55	0.464	0.02	[0.00, 0.08]
	Session × Stimulation	*F*(1, 70.52) = 0.00	0.947	< 0.001	[0.00, 0.01]
Inhibitory control task: reaction time	Session^b^	*F*(1, 69.73) = 5.80	0.019	0.07	[0.01, 0.19]
	Stimulation	*F*(1, 24.78) = 0.20	0.657	0.01	[0.00, 0.06]
	Session × Stimulation	*F*(1, 69.73) = 1.32	0.254	0.02	[0.00, 0.09]
TFEQ Restraint	Assessment (t1)	*t*(34.6) = -0.30	0.768	0.003	[0.00, 0.08]
	Assessment (t2)	*t*(31.6) = -0.78	0.442	0.019	[0.00, 0.05]
	Stimulation	*t*(29.0) = -0.72	0.476	0.018	[0.00, 0.06]
	Assessment × Stimulation (t1)	*t*(35.7) = - 0.25	0.802	0.002	[0.00, 0.08]
	Assessment × Stimulation (t2)	*t*(33.9) = 0.18	0.856	0.001	[0.00, 0.13]
TFEQ Hunger	Assessment (t1)	*t*(27.6) = -2.31	0.028	0.16	[0.00, 0.41]
	Assessment (t2)	*t*(37.2) = -1.03	0.308	0.028	[0.00, 0.20]
	Stimulation	*t*(32.1) = 0.82	0.419	0.020	[0.00, 0.20]
	Assessment × Stimulation (t1)	*t*(28.0) = 0.04	0.965	< 0.001	[0.00, 0.14]
	Assessment × Stimulation (t2)	*t*(35.9) = -1.25	0.220	0.042	[0.02, 0.23]
TFEQ Disinhibition	Assessment (t1)	*t*(34.4) = -0.82	0.419	0.019	[0.00, 0.19]
	Assessment (t2)	*t*(31.1) = -1.00	0.326	0.031	[0.00, 0.23]
	Stimulation	*t*(37.0) = 2.01	0.051	0.10	[0.00, 0.31]
	Assessment × Stimulation (t1)	*t*(32.6) = -0.40	0.695	0.005	[0.00, 0.15]
	Assessment × Stimulation (t2)	*t*(26.3) = -1.08	0.292	0.042	[0.00, 0.27]
FAAQ Acceptance	Assessment (t1)	*t*(30.6) = 0.46	0.650	0.007	[0.00, 0.17]
	Assessment (t2)	*t*(37.5) = 1.13	0.267	0.033	[0.02, 0.21]
	Stimulation	*t*(34.6) = 0.28	0.781	0.002	[0.00, 0.13]
FAAQ Willingness	Assessment (t1)	*t*(34.8) = 1.43	0.162	0.056	[0.01, 0.26]
	Assessment (t2)	*t*(34.6) = 0.76	0.450	0.017	[0.00, 0.18]
	Stimulation	*t*(33.7) = 0.72	0.475	0.015	[0.00, 0.18]

Assessment = t0, t1, t2; FAAQ, Food Craving Acceptance and Action Questionnaire; Stimulation = verum or sham group; TFEQ, Three-Factor-Eating-Questionnaire

^a^ 95% confidence intervals were derived from the noncentral F distribution. Lower bounds of η²_p_ for very small *F-values* that could not be reliably estimated were reported as 0. ^b^ Session was modeled as a continuous predictor (coded 1–6)

### Secondary Outcomes

Exploratory post-hoc tests indicated large-sized decreases in BMI and increases in subjective well-being (WHO-5 total score; Supplementary Table S3) driven by improvements from t0 to t1 and t2 (BMI: t0-t1 and t0-t2, respectively: *t*(30.1) = 5.10, *p* <.001; WHO-5: t0-t1, *t*(26.6) = − 2.93, *p* =.019, t0-t2, *t*(26.1) = − 2.87, *p* =.021). Similarly, exploratory tests suggested large-sized increases in GLP-1 plasma levels resulting from post-hoc increases from t0 to t2, *t*(74.8) = − 3.03, *p* =.009 (Supplementary Figure S1).

## Discussion

This was the first pilot study assessing the feasibility and acceptability of a multimodal intervention after MBS, combining a structured psychosocial intervention led by a psychotherapist and a dietician, and a food-specific ICT enhanced by tDCS. Retention rates were somewhat lower than in a related trial by Giel et al. in binge-eating disorder [42] but within an acceptable range considering reports that patients frequently underrecognize the need for, and thus underutilize, additional support early after MBS [44]. This interpretation is supported by substantial recruitment challenges, with only a minority of screened patients ultimately enrolled, and a large proportion declining participation due to low interest or perceived burden (Fig. 2). Together, these findings point to a mismatch between a clinically critical postoperative window for prevention and a limited patient-perceived need for additional support during this early postoperative phase.

Importantly, however, the intervention itself was well tolerated, with negligible side effects further supporting feasibility. Both verum and sham groups exhibited improvements in inhibitory control, weight loss, hrQOL, and eating behaviors alongside expected changes in endocrine markers. Despite patients’ primary motivation being weight loss, these positive outcomes across psychological and behavioral domains suggest an added benefit of a multiprofessional FU support to optimize the outcomes of MBS [57]. These effects may become particularly relevant long term, as patients often perceive surgical weight reduction alone as insufficient for achieving sustained change and instead value integrative support that emphasizes personal engagement [44, 45, 58–60]. Both the psychoeducational and nutritional components may not only promote health literacy, self-management, long-term adherence and weight maintenance [61] but also simultaneously prevent unrealistic expectations [58, 62, 63].

Our study extends the neuromodulatory research on obesity and eating disorders to the post–bariatric phase [26, 28, 29, 36, 64]. Although postoperative FU care within the first year after MBS is widely recommended from a preventive perspective [3, 47] and was consequently implemented in our intervention, early improvements in response inhibition and mental functioning and well-being [65, 66] may have paradoxically reduced perceived need for additional care [44] and hindered our recruitment process (Fig. 2). Specifically, considering inclusion limited to the early postoperative phase – characterized by pronounced spontaneous improvements – participants who enrolled and completed the study likely represent a self-selected, highly motivated subgroup. This selection may have attenuated intervention effects, as participants may have already implemented substantial behavioral and cognitive changes, leaving limited room for additional benefit, particularly because tDCS appears most effective in individuals with heightened vulnerability to overeating [27].

The lack of differences between active and sham tDCS is consistent with two previous pilot studies that applied single-session TMS or multi-session tDCS prior to MBS [30–33]. Both studies observed stimulation-induced EEG alterations, whereas behavioral effects were absent or minimal, suggesting neural changes may not translate into clinically meaningful behavioral effects in the first months [42]. In the present pilot study, this lack of differential effects between verum and sham groups is likely influenced by the dynamic early postoperative phase, characterized by limited (behavioral) variability. Together with the selective inclusion of a highly motivated sample, this may have masked stimulation-specific effects. Overall, the findings further highlight intervention timing a critical determinant not only for healthcare utilization and potential selection bias, but also for the detectability of neuromodulation effects. The absence of group differences should still be interpreted within the exploratory and feasibility-focused scope of this trial, providing initial estimates of effect sizes and trends to inform adequately powered future studies with extended FU periods.

### Strengths and Limitations

This novel multimodal intervention integrates specialized psychosocial FU care with targeted tDCS and food-specific ICT, providing a first step to bridge unmet treatment needs of patients after MBS. A key strength is the double-blind design, with patients guessing their assigned condition at chance level indicating a powerful control condition. Expectancy-related bias, which is known to strongly affect eating-related outcomes in obesity [67], seems therefore minimized. The use of patient-centered, MBS-specific validated outcome measures, aligned with international core outcome recommendations [68, 69] strengthens clinical relevance. Inclusion of endocrine markers provides unique insights into intervention-related changes in early postoperative gut hormone dynamics.

Several limitations should be acknowledged. The small sample size was based on feasibility rather than statistical power [70, 71]. The absence of neural outcome measures, longitudinal FU assessments and a treatment-as-usual or dismantling condition limits conclusions regarding temporal unfolding and specificity of tDCS-induced effects. Attempts to include data from our in-house psychosocial FU group failed due to poor adherence, a common challenge in post-bariatric populations [72, 73]. The lack of established minimal clinically important differences for MBS-specific instruments [68] restricts clinical interpretation. Implementation during the rapidly changing early postoperative phase and the exclusively female sample likely limited effect detectability, perceived need for support and generalizability.

## Conclusions

Heterogeneous long-term outcomes underscore the need for adjunctive, easily implementable interventions that extend beyond standard surgical and pharmacological care to support sustained lifestyle adaptation. Yet, the present findings suggest that the early postoperative phase may be characterized by a mismatch between low perceived need for additional support and its potential to target early behavioral consolidation processes before dysfunctional eating patterns or weight regain (re-)emerges. Adjunctive interventions during this critical transition phase may therefore appear underutilized, although they may still represent a meaningful contribution to prevention and long-term stabilization. The current study suggests potential for tDCS in the treatment of obesity and specifically after MBS, while further investigation of optimal stimulation parameters, including dose, timing, target regions [26], evaluation of concurrent behavioral tasks and comparison to stimulation modalities like TMS [29] is imperative. Incorporating neurobiological measures may allow earlier detection of effects and help delineate temporal cascades from neural to behavioral and clinical outcomes. Studies with longer FU intervals are needed to clarify whether the early postoperative phase represents a true preventive window and to capture delayed effects, including psychological distress that may emerge once postoperative changes stabilize [59]. Finally, dismantling studies and predictor analyses will be key to identify which intervention components are most effective for which patients, facilitating personalized and scalable implementation in FU treatment after MBS.

## Supplementary Information

Below is the link to the electronic supplementary material.

Supplementary Material 1 (DOCX 288 KB)

## Data Availability

The datasets generated and/or analyzed during the current study due to privacy and ethical restrictions but are available from the corresponding author upon reasonable request. All analysis scripts are publicly available at the Open Science Framework (https://osf.io/4aktp/).
