# Publisher Correction: Enhancing Postoperative Outcomes After Metabolic Bariatric Surgery: a Pilot Study of Inhibitory Control Training, Transcranial Direct Current Stimulation and Psychosocial Aftercare

**DOI:** 10.1007/s11695-026-08886-w

**Published:** 2026-08-12

**Authors:** Sarah Alica Rösch, Carsten Thiele, Therese Reinstaller, Tino Zähle, Kathrin Schag, Katrin Giel, Christian Plewnia, Johann Steiner, Florian Junne, Susanne Vogt

**Affiliations:** 1 University Clinic for Psychosomatic Medicine and Psychotherapy, University Medicine, Otto-von-Guericke-University Magdeburg, Medical Faculty, Magdeburg, Germany; 2 Partner Site Halle-Jena-Magdeburg, German Center for Mental Health (DZPG), Magdeburg, Germany; 3 Department of Neurology, University Medicine, Otto-von-Guericke-University Magdeburg, Magdeburg, Germany; 4 Center for Behavioral Brain Sciences (CBBS), Magdeburg, Germany; 5 Institute for Medical Psychology, Otto-von-Guericke University, Magdeburg, Germany; 6 University Clinic for General, Visceral, Vascular and Transplant Surgery, University Hospital, Otto von Guericke University Magdeburg, Magdeburg, Germany; 7 Partner Site Tübingen, German Center for Mental Health (DZPG), Tübingen, Germany; 8 Department of Psychosomatic Medicine und Psychotherapy, Medical University Hospital Tübingen, Eberhard Karl University Tübingen, Tübingen, Germany; 9 Center of Excellence for Eating Disorders (KOMET), Tübingen, Germany; 10 Department of Psychiatry and Psychotherapy, Medical University Hospital Tübingen, Eberhard Karl University Tübingen, Tübingen, Germany; 11 Department of Psychiatry, University Medicine, Otto-von-Guericke-University Magdeburg, Magdeburg, Germany; 12 Laboratory of Translational Psychiatry, University Hospital, Otto-von-Guericke- University Magdeburg, Magdeburg, Germany; 13 Center for Health and Medical Prevention (CHaMP), Magdeburg, Germany

**Publisher Correction: Obesity Surgery (2026) 36:4316-4327**

**https://doi.org/10.1007/s11695-026-08828-6**

Following publication, errors were identified in the reference numbering. Several references had been omitted, resulting in incorrect numbering of the in-text citations and inconsistencies with the reference list. The reference list and all corresponding in-text citations have been corrected. These changes are limited to the references and do not affect the scientific content, results, interpretation or conclusions of the article.

