# Supplementary material for: Enhancing Postoperative Outcomes After Metabolic Bariatric Surgery: a Pilot Study of Inhibitory Control Training, Transcranial Direct Current Stimulation and Psychosocial Aftercare
Source: Obes Surg. 2026 Jul 15;36(8):4316–27. doi: 10.1007/s11695-026-08828-6 (PMC13429539; doi:10.1007/s11695-026-08828-6)
Supplement: Supplementary file 1 — Supplementary Material 1 (DOCX 288 KB) [file 11695_2026_8828_MOESM1_ESM.docx]

**Online Supplementary Material for:**

# Supplementary Methods

## **Inclusion and Exclusion Criteria**

Adult patients with a body-mass-index (BMI) ≥ 35 kg/m² who underwent sleeve gastrectomy in the past 18 months and who provided written informed consent were included in the study. Patients were excluded if they had language barriers, serious mental or neurological disorders, reported the intake of tranquilizers, neuroleptics, GLP-1-receptor agonists or dopaminergic medication. Patients were also excluded if they reported previous neurosurgical procedures, traumatic brain injury or alcohol or drug abuse, current pregnancy or lactation or an insufficiently adjusted Diabetes mellitus Type 2. Additional exclusion criteria were metal or electric implants in the head (e.g., cochlea implants) or pacemakers.

## **Sleeve Gastrectomy**

During the sleeve gastrectomy, after gastric mobilization, a 36 Fr bougie was positioned abutting the lesser curvature. 6 cm proximal to the pylorus the greater curvature was resected using a laparoscopic linear stapling device, following lateral alongside the bougie from the antrum to the angle of His with complete resection of the fundus, creating a narrow, tube-shaped sleeve. The staple line was not reinforced.

## **Use of Artificial Intelligence Tools**

The first author used ChatGPT (OpenAI, GPT-5.3) as a supportive tool for improving language clarity and coherence. The tool was also consulted to assist with identifying appropriate R commands and troubleshooting code during data analysis. The AI system did not contribute to the study design, data analysis decisions, or interpretation of results. All content was critically reviewed and approved by the first author, who takes full responsibility for the integrity and accuracy of the work.

## **Measures**

### Secondary Outcomes

Food-related cravings were determined through the 15 items of the Food Cravings Questionnaire-Trait-reduced FCQ-T-r [1,2]. Subjective well-being over the past 2 weeks were quantified through the 5 items of the WHO-5 well-being index [3]. Regarding eating behavior, the Eating Disorder Examination Interview – Bariatric Surgery Version (EDE-BSV; [4], conducted by the first author as a licensed psychotherapist, was used to measure *restraint*, *eating concern*, *weight concern*, and *shape concern* as well as the surgery-specific scales *plugging* and *dumping* over the last 4 weeks. In addition, the Eating Disorder Examination Questionnaire (EDE-Q; [5–7]), composed of six diagnostic items and 22 items on *restraint*, *eating concern*, *weight concern*, and *shape concern* over the last 28 days was administered. The Quality of Life for Obesity Surgery (QOLOS; [8]) Questionnaire was used to measure health-related quality of life with 36 pre-and postoperative items on *eating disturbances*, *physical functioning*, *body satisfaction*, *family support*, *social discrimination*, *positive activities* and *partnership*. The QOLOS includes 20 further items on *excess skin*, *eating adjustment*, *dumping* and *satisfaction with surgery* which are only administered post-operatively. Turning to general impulsivity, the short version of the Barratt Impulsiveness Scale (BIS-15; [9,10]) depicted *non-planning impulsivity, motor impulsivity* and *attentional impulsivity* via 15 items, and the Impulsive Behavior-Scale (UPPS; [11]), composed of 40 items, measured *negative urgency*, *lack of premeditation*, *positive urgency* and *lack of perseverance*. A self-developed protocol was additionally used to assess common impulsive behaviors within the last seven days [12]. Based on the Diagnostic and Statistical Manual of Mental Disorders, Fifth Version [13] and the International Classification of Diseases, Tenth Revision [14], the structured diagnostic interview Mini-DIPS (Diagnostisches Kurz-Interview bei psychischen Störungen; [15]) depicted mental disorder comorbidity. General psychopathology and depressive symptoms were measured through the 21 items of the Beck Depression Inventory (BDI II; [16]) and the 59 items of the Patient Health Questionnaire (PHQ-D; [17,18]), depicting *depressive symptoms*, *somatic symptoms*, *anxiety symptoms*, *eating disorder symptoms* and *alcohol-related disorders*.

## Endocrine Markers

To prevent enzymatic hormone degradation, aprotinin was inserted into ethylenediaminetetraacetic acid tubes (500 KIU per ml blood). Tubes were cooled at a temperature of 4°C before centrifugation, which was performed at the same temperature for 15 min with a Relative Centrifugal Force of 1600g. Plasma aliquotes of 0.5 ml Protein LoBind Tubes (Eppendorf) were subsequently kept at -80°C. Glucagon-like Peptide 1 (GLP-1), peptide YY (PYY) and total Ghrelin (Merck, Human PYY Enzyme-linked Immunosorbent Assay [ELISA] Kit:#RAB1078-1KT; GLP-1 Enzyme Immunoassay [EIA] Kit:#RAB0201-1KT; Ghrelin EIA Kit:# RAB0207-1KT) were measured through duplicate ELISA and EIA-Kits.

## Group Intervention

Patients participated in monthly psychosocial group sessions guided by a licensed psychologist and a registered dietitian. Group contents were based on cognitive-behavioral and psychodynamic principles and focused on behavioral, emotional, and lifestyle adaptation (e.g., dealing with supplements, physical activity, social support, and management of high-risk situations like vacation). The first session was scheduled to approximately coincide with the first transcranial direct current stimulation (tDCS) session. The group operated in a semi-open format due to ongoing enrollment, allowing new patients to join at any time.

## Data Processing

Data were first pseudonymized and stored according to the European General Data Protection Regulation 2018. A research assistant entered data manually from paper versions to an electronic study database. Fidelity and plausibility of the data were checked by the first author. For questionnaire analysis, when at least 50% of the items were completed, the sum of valid items was scaled up to the full number of items. Otherwise, the score was set to missing, following the half-scale rule [19]. Group differences in sample characteristics, success of masking, and potential side effects were calculated with *t* tests for independent samples, χ2 tests or Mann Whitney U Tests. Test prerequisites (e.g., assumption of normality or variance homogeneity) were validated with appropriate tests (e.g., Shapiro-Wilk, Levene’s test). Side effects associated assessed across seven factors (e.g., tingling in the area of the electrode, fatigue, slight itching) were aggregated into a mean side effect index. Internal consistency of questionnaire scales was assessed using Cronbach’s α at t0.

### Inhibitory Control Training

Eye-tracking data initially underwent rigorous preprocessing to ensure data quality. Trials with premature saccades, defined as < 80 ms, or delayed saccades, defined as > 900 ms or missing responses, were excluded [20]. Entire sessions were excluded if more than 25% of the trials were invalid or in the case of technical errors (e.g., eye tracker malfunction). In total, 14 (13.59%) datasets were excluded from analyses (13 due to bad data quality, one due to technical problems during all sessions with one person). Since missing data resulted exclusively from deliberate quality exclusions due to technical recording failures, it could not be assumed to be missing at random and multiple imputations were not performed.

### Endocrine Markers

PYY concentrations were not analyzed due to technical difficulties with the commercially available assay kits. Across multiple kit batches, the assays failed to generate reliable standard curves, rendering the resulting measurements invalid. Given the lack of reproducible results and the limited availability of plasma samples, further analyses were discontinued.

To achieve a normal distribution, ghrelin and GLP-1 values were log-transformed. Sensitivity analyses excluded laboratory values with a coefficient of variation > 10%, which affected 14 out of 104 datasets (13.46%).

## Sample Size

Given its nature as a clinical pilot study targeting feasibility and effect sizes instead of statistical significance, and the lack of studies applying tDCS in postbariatric samples, a sample size calculation was not conducted.

## **Statistical Analyses**

Linear mixed-effects models (LMMs) accounted for the hierarchical data structure and allowed for the use of all available observations without requiring a complete data set across assessment points for each participant.

For questionnaire-based primary outcomes (Three Factor Eating Questionnaire, Food Craving Acceptance and Action Questionnaire), missing data were handled using multiple imputation at the scale level. Twenty imputed datasets were generated using predictive mean matching. Age, stimulation condition, and baseline scores were included as predictors in the imputation model. Sex was not included due to the exclusively female sample contrary to the original plan [21]. LMMs were fitted separately within each imputed dataset, and fixed-effect estimates were pooled according to Rubin’s rules, incorporating both within- and between-imputation variance. Because pooling of Type-III ANOVA tables was not directly supported under Rubin’s framework, statistical inference for questionnaire outcomes was based on pooled fixed-effect estimates.

For eyetracking data, mean reaction times (from correct trials) and error rates (percentage of incorrect trials) were computed for each patient and session. These aggregated data were analyzed using complete-case LMMs, with session number (treated as a numeric variable, 1–6) and stimulation group (verum vs. sham) and their interaction included as fixed effects.

For endocrine markers, plasma hormone concentrations were analyzed using LMMs with assessment (t0, t1, t2), measurement (fasted, 30 min), stimulation group (verum vs. sham), and all interactions included as fixed effects.

### Effect Sizes

Partial eta squared (η²ₚ) was calculated for each fixed effect based on the corresponding pooled test statistic. Depending on the model component, Satterthwaite or Kenward–Roger approximations were used to determine the degrees of freedom. For fixed effects evaluated via *t*-tests, η²ₚ was computed as:

$$\eta_{p}^{2}=\frac{t^{2}}{t^{2}+df}$$

where $t$is the Satterthwaite-corrected t-value and $df$is the corresponding degrees of freedom. This provides an estimate of the degree of variance explained by each fixed effect relative to residual variance, which is recommended when traditional ANOVA-based η²ₚ is not directly available in LMMs [22,23].

### Post-hoc Analyses

Post-hoc pairwise comparisons were conducted using estimated marginal means via the emmeans package [24]. For significant main effects only, Tukey-adjusted pairwise contrasts were computed exploratively and reported descriptively.

# Supplementary Results

## **Primary Outcomes: Feasibility and Acceptance**

On self-developed items, satisfaction with program length (*M* = 3.20, *SD* = 0.56) and session duration (*M* = 3.12, *SD* = 0.34; 1 = *far too short*, 5 = *far too long*) was moderate-high. No differences between stimulation groups emerged regarding perceived improvements in eating behavior, *W* = 39.5, *p* = .418, and impulse control, *W* = 38, *p* = .535. In free-text responses for side effects, *n* = 1 patient of the verum group reported increased concentration, *n* = 1 patient of the sham group temporary numbness in arms or hands and another patient of the sham group a brief metallic taste at the onset of stimulation.

Among verum patients, mean confidence was 63.8% (*SD* = 11.1) for those guessing active stimulation and 36.7% (*SD* = 37.9) for those guessing sham. Among sham patients, mean confidence was 86% (*SD* = 11.4) for those guessing active and 66.7% (*SD* = 15.3) for those guessing sham stimulation. Patients in the sham group were significantly more confident that they had received active stimulation (*M* = 86%, *SD* = 11.4) than patients in the verum group (*M* = 63.8%, *SD* = 11.1), Welch’s *t*-test, *t*(6.65) = –2.95, *p* = .023, 95% CI [–40.25, –4.25]. Although patients could not reliably identify their stimulation condition overall, those in the sham group tended to be more confident when incorrectly guessing they had received active stimulation.

Patients reported high overall satisfaction with the stimulation device during training sessions (*M* = 4.06, *SD* = 0.77, 1 = *not at all satisfied*, 5 = *very satisfied*). Technical problems with the stimulation device were only reported by one patient in the verum group and not further specified by the patient; probably referring to the stimulation device not working for this patient in two sessions.

Patients evaluated the perceived usefulness of brain stimulation for improving impulse control (*M* = 3.44, *SD* = 1.15; median = 4; 1 = *not useful at all*, 5 = *very useful*) and for improving eating behavior as moderate-high (*M* = 3.12, *SD* = 1.20; median = 3.5). Patients reported the device as rather easy to use (*M* = 3.62, *SD* = 0.89; median = 4, 1 = *very complicated*, 5 = *very easy*). Responses regarding future brain stimulation were mixed (*M* = 2.31, *SD* = 1.20; median = 2; 1 = *yes*, 5 = *no*).

Qualitative free-text responses provided further insight into patients’ experiences. Positive aspects of the stimulation referred to the innovative nature of the intervention (*n* = 1), perceived increases in concentration or alertness (*n* = 2), low physical sensation during stimulation (*n* = 3; e.g., “not noticeable,” “not painful”), and low overall burden (*n* = 1). Negative aspects primarily concerned the duration of stimulation sessions (*n* = 2), discomfort related to electrode application materials such as adhesive or gel (*n* = 2), or practical inconveniences (*n* = 1). Several patients explicitly stated that they had no complaints (*n* = 3). Suggestions for improvement were sparse and primarily focused on minor optimizations, such as adapting visual stimuli (*n* = 1), improving the method of electrode placement (e.g., alternative headgear; *n* = 1), or indicated no need for changes (*n* = 4). Additional comments included general reflections on the subjectivity of self-report measures (*n* = 1), uncertainty about assessing impulse control changes at an early postoperative stage (*n* = 1), and suggestions to enhance stimulation effects by combining sensory modalities (e.g., visual stimuli with food-related odors; *n* = 1).

## **Secondary Outcomes**

None of the subscale-level (i.e., subscales of the EDE, EDE-Q, QOLOS, BIS-15, PHQ-D) analyses yielded statistically significant effects (all *p*s > .05). Detailed results will be reported in secondary analyses and remain hitherto available only upon request.

For log-transformed ghrelin concentrations, a significant main effect of assessment was observed, *F*(2, 75.11) = 3.25, *p* = .044, η²ₚ = .08, 95% CI [.00, .20] (Supplementary Table S3 and Figure S1). Exploratory Tukey-adjusted pairwise contrasts, however, did not reveal statistically significant differences between t0 and t1, *t*(75.2) = −2.05, *p* = .107, t0 to t2, *t*(75.4) = −2.32, *p* = .059, or t1 and t2, *t*(74.2) = −0.32, *p* = .947 and the main effect of assessment was no longer statistically significant in sensitivity analyses, *F*(2, 61.56) = 2.16, *p* = .124, η²ₚ = .066, 95% CI [.00, .20].

For log-transformed GLP-1 concentrations, the significant main effect of assessment remained robust in sensitivity analyses, *F*(2, 60.66) = 4.87, *p* = .011, η²ₚ = .14, 95% CI [.01, .29]. Exploratory Tukey-adjusted pairwise contrasts indicated a significant increase in GLP-1 levels from t0 to t2, *t*(61.5) = −3.05, *p* = .009, while the comparison between t0 and t1, *t*(61.2) = −2.12, *p* = .095, and t1 and t2, *t*(60.2) = −0.92, *p* = .631, did not reach statistical significance. In summary, sensitivity analyses for endocrine markers corroborated observed assessment-related increase in GLP-1 levels particularly between t0 and t2, whereas the assessment effect for ghrelin appeared less robust after outlier exclusion.

Turning to the EDE-BSV, weight-related vomiting was reported infrequently overall and occurred more often in the sham group (Supplementary Table S4). However, given the very small number of cases and the limited sample size, these findings should be interpreted with considerable caution.

# Supplementary References

1. Pudel V, Westenhöfer J. Fragebogen zum Essverhalten (FEV). Göttingen: Hogrefe; 1989.

2. Stunkard AJ, Messick S. The three-factor eating questionnaire to measure dietary restraint, disinhibition  and hunger. J Psychosom Res. 1985;29(1):71–83. doi:10.1016/0022-3999(85)90010-8 PubMed PMID: 3981480.

3. Juarascio A, Forman E, Timko CA, Butryn M, Goodwin C. The development and validation of the food craving acceptance and action questionnaire (FAAQ). Eat Behav. 2011;12(3):182–7. doi:10.1016/j.eatbeh.2011.04.008 PubMed PMID: 21741015.

4. Meule A, Hermann T, Kübler A. A short version of the Food Cravings Questionnaire-Trait: the FCQ-T-reduced. Front Psychol. 2014;5(190). doi:https://doi.org/10.3389/fpsyg.2014.00190

5. Cepeda-Benito A, Gleaves DH, Williams TL, Erath SA. The development and validation of the state and trait food-cravings questionnaires. Behav Ther. 2000;31(1):151–73.

6. Brähler E, Zenger M, Kemper CJ. Psychologische und sozialwissenschaftliche Kurzskalen: Standardisierte Erhebungsinstrumente für Wissenschaft und Praxis. MWV Medizinisch Wissenschaftliche Verlagsgesellschaft mbH & Co. KG; 2015.

7. de Zwaan M, Hilbert A, Swan-Kremeier L, Simonich H, Lancaster K, Howell LM, et al. Comprehensive interview assessment of eating behavior 18-35 months after gastric bypass surgery for morbid obesity. Surgery for Obesity and Related Diseases. 2010;6(1):79–85. doi:10.1016/j.soard.2009.08.011 PubMed PMID: 19837012.

8. Fairburn CG, Beglin SJ. Assessment of eating disorders: Interview or self-report questionnaire? Int J Eat Disord. 1994;16:363–370.

9. Fairburn CG, Beglin SJ. Eating Disorder Examination-Questionnaire (Edition 6.0). 2008.

10. Hilbert A, Tuschen-Caffier B. Eating Disorder Examination-Questionnaire: Deutschsprachige Übersetzung. 2. Aufl,. Tübingen: dgvt; 2016.

11. Müller A, Crosby RD, Selle J, Osterhus A, Köhler H, Mall JW, et al. Development and evaluation of the Quality of Life for Obesity Surgery (QOLOS) questionnaire. Obes Surg. 2018;28(2):451–63. doi:10.1007/s11695-017-2864-6 PubMed PMID: 28791603.

12. Meule A, Vögele C, Kübler A. Psychometrische Evaluation der deutschen Barratt Impulsiveness Scale – Kurzversion (BIS-15). Diagnostica. 2011 Jul;57(3):126–33. doi:10.1026/0012-1924/a000042

13. Spinella M. Normative data and a short form of the Barratt Impulsiveness Scale. Int J Neurosci. 2007 Mar;117(3):359–68. doi:10.1080/00207450600588881 PubMed PMID: 17365120.

14. Schmidt RE, Gay P, D’Acremont M, Van der Linden M. A German adaptation of the UPPS Impulsive Behavior Scale: Psychometric properties and factor structure. Swiss Journal of Psychology / Schweizerische Zeitschrift für Psychologie / Revue Suisse de Psychologie. 2008;67(2):107–12. doi:10.1024/1421-0185.67.2.107

15. Giel KE, Schag K, Max SM, Martus P, Zipfel S, Fallgatter AJ, et al. Inhibitory control training enhanced by transcranial direct current stimulation to reduce binge eating episodes: findings from the randomized phase II ACCElect trial. Psychother Psychosom. 2023 May 1;92(2):101–12. doi:10.1159/000529117 PubMed PMID: 36889293.

16. American Psychiatric Association. Diagnostic and statistical manual of mental disorders: DSM-5. 5th edition. Arlington, VA; 2013.

17. World Health Organization. The ICD-10 classification of mental and behavioural disorders. 2nd ed. Geneva PP - Geneva: World Health Organization; 1993.

18. Margraf J, Cwik JC, Pflug V, Schneider S. Strukturierte klinische Interviews zur Erfassung psychischer Störungen über die Lebensspanne: Gütekriterien und Weiterentwicklungen der DIPS-Verfahren [Structured clinical interviews for mental disorders across the life span: Psychometric quality and furt. Z Klin Psychol Psychother. 2017;46:176–86.

19. Hautzinger M, Keller F, Kühner C. BDI-II. Beck Depressions-Inventar. Revision. 2. Auflage. Frankfurt: Pearson Assessment; 2009.

20. Gräfe K, Zipfel S, Herzog W, Löwe B. Screening psychischer Störungen mit dem “Gesundheitsfragebogen für Patienten (PHQ-D)”. Ergebnisse der Deutschen Validierungsstudie. Diagnostica. 2004;50(4):171–81. doi:10.1026/0012-1924.50.4.171

21. Spitzer RL, Kroenke K, Williams JB. Validation and utility of a self-report version of PRIME-MD: the PHQ primary care study. Primary Care Evaluation of Mental Disorders. Patient Health Questionnaire. JAMA. 1999 Nov;282(18):1737–44. doi:10.1001/jama.282.18.1737 PubMed PMID: 10568646.

22. Blechert J, Meule A, Busch NA, Ohla K. Food-pics: An image database for experimental research on eating and appetite. Front Psychol. 2014;5. doi:10.3389/fpsyg.2014.00617

23. Jasper HH. The ten-twenty electrode system of the International Federation. Electroencephalogr Clin Neurophysiol. 1958;10:371–5.

24. Reinhart RMG, Cosman JD, Fukuda K, Woodman GF. Using transcranial direct-current stimulation (tDCS) to understand cognitive processing. Atten Percept Psychophys. 2017;79(1):3–23. doi:10.3758/s13414-016-1224-2 PubMed PMID: 27804033.

25. Kessler SK, Turkeltaub PE, Benson JG, Hamilton RH. Differences in the experience of active and sham transcranial direct current stimulation. Brain Stimul. 2012;5(2):155–62. doi:10.1016/j.brs.2011.02.007 PubMed PMID: 22037128.

26. Fayers P, & MD. Quality of Life: The Assessment, Analysis and Interpretation of Patient-Reported Outcomes. 3rd edition. Wiley; 2016.

27. Giel KE, Schag K, Max SM, Martus P, Zipfel S, Fallgatter AJ, et al. Inhibitory control training enhanced by transcranial direct current stimulation to reduce binge eating episodes: findings from the randomized phase II ACCElect trial. Psychother Psychosom. 2023 May 1;92(2):101–12. doi:10.1159/000529117 PubMed PMID: 36889293.

28. Rösch SA, Wünsche L, Thiele C, Reinstaller T, Zähle T, Schag K, et al. Enhancing the outcomes of bariatric surgery with inhibitory control training, electrical brain stimulation and psychosocial aftercare: a pilot study protocol. J Eat Disord. 2024 Dec 9;12(1). doi:10.1186/s40337-024-01160-3

29. Lakens D. Calculating and reporting effect sizes to facilitate cumulative science: A practical primer for t-tests and ANOVAs. Front Psychol. 2013;4:863. doi:10.3389/fpsyg.2013.00863 PubMed PMID: 24324449.

30. Cohen J. Statistical power analysis for the behavioral sciences (2nd ed.). Hillsdale, NJ: Lawrence Erlbaum Associates.; 1973.

31. Lenth R. emmeans: Estimated Marginal Means, aka Least-Squares Means (Version 1.8.5). https://CRAN.R-project.org/package=emmeans; 2023.

# Supplementary Figures

## Supplementary Figure S1

*Results for log-transformed Endocrine Markers*

| 1. 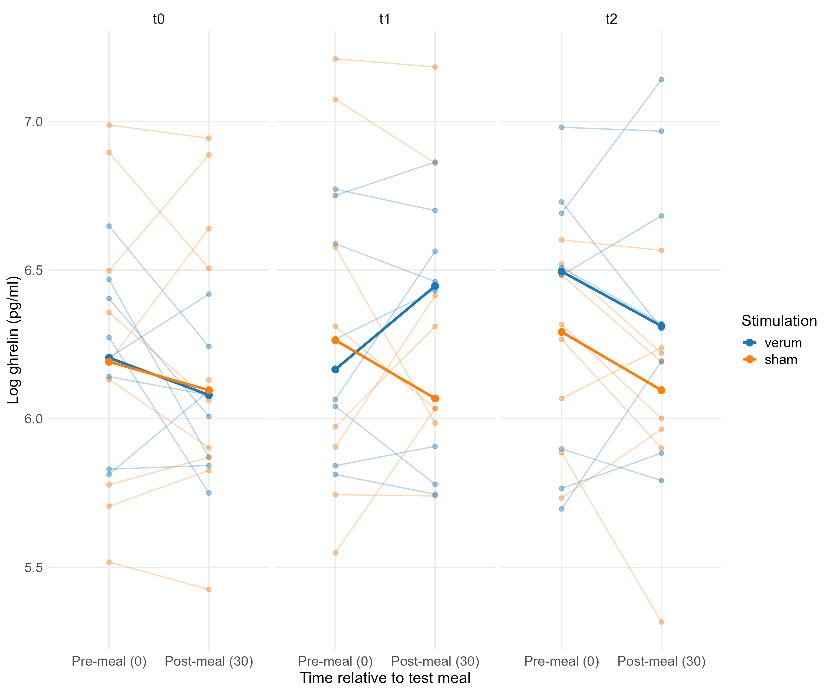 | 1. 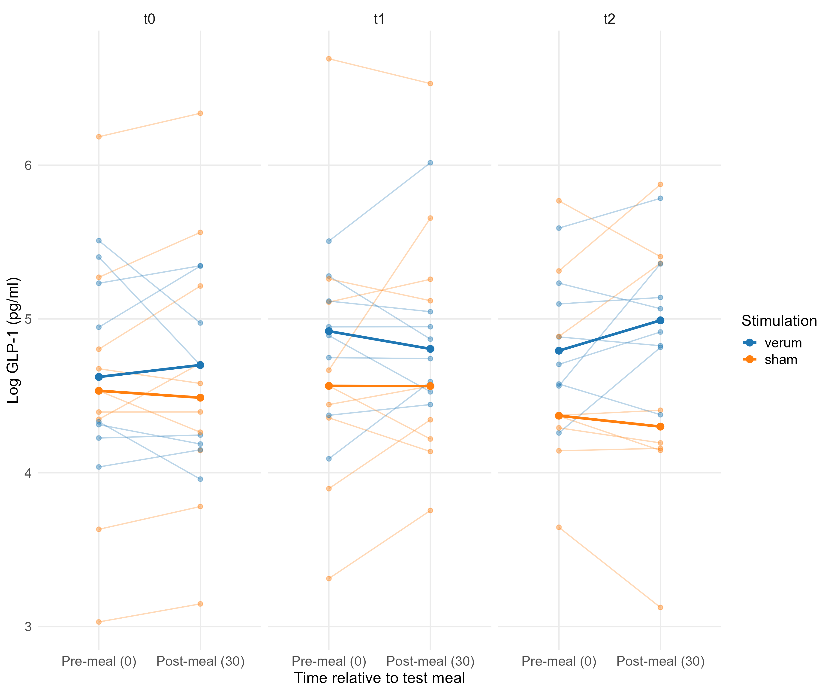 |
| --- | --- |

*Note*. Boxplots of log-transformed (A) ghrelin or (B) GLP-1 levels measured immediately before (0 min) and 30 minutes after consumption of a standardized liquid test meal, stratified by assessment (t0, t1, t2). Thin lines represent individual patients; thick lines indicate group medians.

# Supplementary Tables

## Supplementary Table S1

*Assessment Points and Instruments*

| Measure | Instrument | | | t0 | | | t1 | | t2 | α^a^ |
| --- | --- | --- | --- | --- | --- | --- | --- | --- | --- | --- |
| Sociodemographic data |  | | | x | | |  | |  |  |
| Primary Outcomes |  | | |  | | |  | |  |  |
| Feasibility and acceptance | - % included from eligible patients at t0, - drop-out rate through t0 to t2 - self-developed questionnaire - side effects of tDCS | | |  | | | x | |  |  |
| Food-specific impulsivity | error rate and latency in the inhibitory control training | | | x | | | x | | x |  |
|  | TFEQ Restraint | | | x | | | x | | x | .79 |
|  | TFEQ Hunger | | | x | | | x | | x | .85 |
|  | TFEQ Disinhibition | | | x | | | x | | x | .80 |
|  | FAAQ Acceptance | | | x | | | x | | x | .82 |
|  | FAAQ Willingness | | | x | | | x | | x | .86 |
| Secondary Outcomes |  | | |  | | |  | |  |  |
| Eating behavior-related outcomes |  | | |  | | |  | |  |  |
| - Eating disorder psychopathology | EDE-BSV | | | x | | | x | | x |  |
| - Eating disorder psychopathology | EDE-Q | | | x | | | x | | x | .84 |
| - Food-related cravings | FCQ-T-r | | | x | | | x | | x | .97 |
| - Height and weight to compute | BMI (kg/m²) | | | x | | | x | | x |  |
| Health-related quality of life (HrQOL): |  | | |  | | |  | |  |  |
| - HrQOL before and after MBS | Section 1 QOLOS total score | | | x | | | x | | x | .91 |
|  | Section 1 QOLOS total score | | | x | | | x | | x | .79^b^ |
| - Quality of life | WHO-5 | | | x | | | x | | x | .89 |
| Impulsivity-related outcomes |  |  |  | |  |  | |  |  |  |
| - General impulsivity | BIS-15 | | | x | | | x | | x | .73 |
| - General impulsivity | UPPS | | | x | | | x | | x | > .72 |
| - General impulsivity | Self-developed protocol | | | x | | | x | | x |  |
| General psychopathology |  | | |  | | |  | |  |  |
| - Mental disorder comorbidity | Mini-DIPS | | | x | | | x | | x |  |
| - Depressive symptoms | BDI | | | x | | | x | | x | .89 |
| - Depressive symptoms | PHQ-D | | |  | | |  | |  | .74 |
| Endocrine markers |  | | | x | | | x | | x |  |
| - Secretion of ghrelin |  | | | x | | | x | | x |  |
| - Secretion of PYY 1 |  | | | x | | | x | | x |  |
| - Secretion of GLP-1 |  | | | x | | | x | | x |  |

*Note*. BDI, Beck Depression Inventory; BIS-15, short version of the Barratt Impulsiveness Scale; EDE-BSV, Eating Disorder Examination Interview – Bariatric Surgery Version; EDE-Q, Eating Disorder Examination Questionnaire; FAAQ, Food Craving Acceptance and Action Questionnaire; FCQ-S, Food Cravings Questionnaire-State; FCQ-T, Food Cravings Questionnaire-Trait-reduced; GLP-1, glucagon like peptide-1; MBS, metabolic bariatric surgery; Mini-DIPS, Diagnostisches Kurz-Interview bei psychischen Störungen; PHQ-D, Patient Health Questionnaire; PYY, peptide YY; QOLOS, Quality of Life for Obesity Surgery; t0, baseline assessment after the sleeve gastrectomy; t1, after the tDCS intervention; t2, after the psychosocial intervention; TFEQ, Three Factor Eating Questionnaire; UPPS, Impulsive Behavior-Scale; WHO-5, well-being index.
^a^For all questionnaires, cronbach’s α reflects internal consistency at t0 only and was reported in a standardized manner. As α was low or could not be reliably estimated for some subscales due to restricted item variance and the small sample size; results should be interpreted cautiously. For instance, all patients answered the item “I regret that I had bariatric surgery done” corresponding to the Section 1 QOLOS subscale “surgery satisfaction” with 5 = completely not true. Some items (BDI item *Thoughts of killing myself*, PHQ Depression item *Thoughts that you would be better off dead or of hurting yourself in some way*) showed no variance at t0 and were excluded from the reliability estimation.

## Supplementary Table S2

*Baseline Characteristics*

|  | Verum group (*n =* 9) | Sham group (*n =* 10) | *Test statistics* |
| --- | --- | --- | --- |
| Age (*M*, *SD*) | 54.04 (11.73) | 46.44 (15.92) | *t*(16.41) = 1.19, *p =*.249 |
| Biological female sex (*N*, %) | 9 (100%) | 10 (100%) |  |
| Time since surgery (*M*, *SD*) | 0.61 (0.47) | 0.75 (0.69) | *t*(15.96) = -0.50, *p =*.624 |
| BMI (*M*, *SD*) | 43.22 (6.31) | 46.09 (8.88) | *t*(16.19) = -0.82, *p =*.426 |
| Education, % (*n*)^1^ |  |  | Fisher’s exact test, *p* = .462 |
| ≥ 12 years | 3 (33%) | 3 (30%) |  |
| Employment^1^ |  |  |  |
| Full-time | 0 | 2 (10%) |  |
| Part-time 15 – 35h/week | 1 (11%) | 0 |  |
| Part-time 15h/week | 7 (78%) | 5 (50%) |  |
| Unemployed | 1 (11%) | 2 (20%) |  |
| Retired | 0 | 1 (10%) |  |
| Income of all household members^1^ |  |  | Fisher’s exact test, *p* = 1.00 |
| < 1.500 € | 6 (67%) | 6 (60%) |  |
| 1.500 – 3000 € | 1 (11%) | 1 (10%) |  |
| > 3.000 € | 1 (11%) | 2 (20%) |  |
| Declined to report | 1 (11%) | 1 (10%) |  |
| Number of somatic comorbidities | 3.89 (2.15) | 4.6 (2.07) | *t*(16.62) = -0.73, *p =* 0.473 |
| Psychotherapeutic treatment (*N*, %) | 0 | 2 (20%)^2^ |  |
| Motivation to change eating behavior^1^ (*M*, *SD*) | 9 (1.2) | 9.56 (0.73) | *W =* 27, *p =* .357 |
| Readiness to change eating behavior^1^ (*M*, *SD*) | 9.12 (1.13) | 9.33 (0.87) | *W =* 33, *p =* .792 |
| Confidence to change eating^1^ behavior (*M*, *SD*) | 8.25 (1.39) | 9.00 (1.00) | *t*(12.60) = -1.26, *p =* 0.229 |
| Time between t0 and t1 in days (*M*, *SD*) | 70.75 (9.91) | 71.67 (18.83) | *t*(12.4) = -0.128, *p* = .901 |
| Time between t0 and t2 in days (*M*, *SD*) | 125.88 (12.7) | 125.75 (16.12) | *W =* 37, *p =* .634 |
| Time between t1 and t2 in days (*M*, *SD*) | 55.12 (12.41) | 54 (19.91) | *t*(11.73) = 0.135, *p =* .894) |
| Mean number of group sessions at t1 (Median) | 1 | 1 | W = 44.5, p = .738 |
| Mean number of group sessions at t2 (Median) | 2 | 2.5 | W = 36.5, *p* = 1.0 |

*Note*. ^1^Data is missing for *n =* 1 patient in each group for questionnaire data. ^2^ Both patients reported psychotherapeutic treatment unrelated to BS. One of these two patients reported concurrent intake of Escitalopram.

## Supplementary Table S3

*Effects of Group and Assessment on Secondary Outcomes*

| Outcome | Effect | Test Statistics | *p* | η²ₚ^a^ | 95% CI η²ₚ ^b^ |
| --- | --- | --- | --- | --- | --- |
| EDE Global | Assessment | *F*(2, 30.54) = 1.72 | 0.195 | 0.10 | [.00, .31] |
|  | Stimulation | *F*(1, 17.17) = 1.22 | 0.284 | 0.07 | [.00, .36] |
|  | Assessment × Stimulation | *F*(2, 30.54) = 0.64 | 0.536 | 0.04 | [.00, .21] |
| EDE-Q Global | Assessment | *F*(2, 32.65) = 1.41 | .259 | .079 | [.00, .27] |
|  | Stimulation | *F*(1, 17.20) = 0.49 | .494 | .028 | [.00, .29] |
|  | Assessment × Stimulation | *F*(2, 32.64) = 0.46 | .636 | .027 | [.00, .17] |
| FCQ-T Total | Assessment | *F*(2, 25.84) = 0.25 | .781 | .019 | [.00, .16] |
|  | Stimulation | *F*(1, 17.26) = 0.54 | .472 | .030 | [.00, .30] |
|  | Assessment × Stimulation | *F*(2, 25.82) = 1.10 | .348 | .079 | [.00, .29] |
| BMI | Assessment | *F*(2, 30.11) = 17.39 | < .001 | .536 | [.26, .69] |
|  | Stimulation | *F*(1, 17.04) = 0.69 | .419 | .039 | [.00, .31] |
|  | Assessment × Stimulation | *F*(2, 30.11) = 0.00 | .999 | < .001 | — |
| WHO-5 | Assessment | *F*(2, 27.07) = 3.43 | .047 | .202 | [.00, .43] |
|  | Stimulation | *F*(1, 17.87) = 0.45 | .513 | .024 | [.00, .28] |
|  | Assessment × Stimulation | *F*(2, 27.05) = 1.38 | .268 | .093 | [.00, .31] |
| Section 1 QOLOS Total Score | Assessment | *F*(2, 23.79) = 1.07 | .261 | .107 | [.00, .34] |
|  | Stimulation | *F*(1, 17.09) = 1.93 | .182 | .102 | [.00, .40] |
|  | Assessment × Stimulation | *F*(2, 23.73) = 0.003 | .966 | .003 | [.00, .02] |
| Section 2 QOLOS Total Score | Assessment | *F*(2, 27.57) = 0.46 | .634 | .033 | [.00, .20] |
|  | Stimulation | *F*(1, 15.85) = 0.85 | .370 | .051 | [.00, .35] |
|  | Assessment × Stimulation | *F*(2, 27.51) = 0.28 | .673 | .028 | [.00, .19] |
| BIS-15 Total | Assessment | *F*(2, 25.50) = 0.21 | .816 | .016 | [.00, .15] |
|  | Stimulation | *F*(1, 17.02) = 0.24 | .633 | .014 | [.00, .26] |
|  | Assessment × Stimulation | *F*(2, 25.49) = 0.58 | .566 | .044 | [.00, .23] |
| UPPS Urgency | Assessment | *F*(2, 24.21) = 0.22 | .808 | .017 | [.00, .16] |
|  | Stimulation | *F*(1, 16.91) = 0.05 | .823 | .003 | [.00, .19] |
|  | Assessment × Stimulation | *F*(2, 24.29) = 0.31 | .739 | .025 | [.00, .18] |
| UPPS Premeditation | Assessment | *F*(2, 25.16) = 1.33 | .282 | .096 | [.00, .32] |
|  | Stimulation | *F*(1, 16.19) = 0.56 | .465 | .033 | [.00, .31] |
|  | Assessment × Stimulation | *F*(2, 25.25) = 0.73 | .492 | .055 | [.00, .25] |
| UPPS Perseverance | Assessment | *F*(2, 25.49) = 0.11 | .901 | .008 | [.00, .10] |
|  | Stimulation | *F*(1, 17.25) = 4.86 | .041 | .220 | [.00, .51] |
|  | Assessment × Stimulation | *F*(2, 25.58) = 0.08 | .924 | .006 | [.00, .08] |
| UPPS Sensation Seeking | Assessment | *F*(2, 23.57) = 0.19 | .831 | .016 | [.00, .15] |
|  | Stimulation | *F*(1, 16.89) = 0.01 | .905 | .001 | [.00, .14] |
|  | Assessment × Stimulation | *F*(2, 23.62) = 0.12 | .890 | .010 | [.00, .12] |
| BDI Total | Assessment | *F*(2, 25.27) = 0.02 | .977 | .002 | [.00, .00] |
|  | Stimulation | *F*(1, 17.44) = 2.45 | .136 | .123 | [.00, .42] |
|  | Assessment × Stimulation | *F*(1, 74.21) = 0.85 | .715 | .026 | [.00, .19] |
| Ghrelin pg/ml (log) | Measurement | *F*(1, 60.48) = 0.36 | .551 | .006 | [.00, .10] |
|  | Stimulation | *F*(1, 16.75) = 0.06 | .813 | .003 | [.00, .20] |
|  | Assessment | *F*(2, 61.56) = 2.16 | .124 | .066 | [.00, .20] |
|  | Measurement × Stimulation | *F*(1, 60.48) = 0.39 | .535 | .006 | [.00, .10] |
|  | Measurement × Assessment | *F*(2, 60.37) = 0.10 | .901 | .003 | [.00, .05] |
|  | Stimulation × Assessment | *F*(2, 61.56) = 2.20 | .120 | .067 | [.00, .20] |
|  | Measurement × Stimulation × Assessment | *F*(2, 60.37) = 1.57 | .217 | .049 | [.00, .17] |
| GLP-1 pg/ml (log) | Measurement | *F*(1, 60.02) = 1.49 | .228 | .024 | [.00, .14] |
|  | Stimulation | *F*(1, 16.64) = 0.09 | .766 | .005 | [.00, .22] |
|  | Assessment | *F*(2, 60.66) = 4.87 | .011 | .138 | [.01, .29] |
|  | Measurement × Stimulation | *F*(1, 60.02) = 0.31 | .580 | .005 | [.00, .09] |
|  | Measurement × Assessment | *F*(2, 59.96) = 0.22 | .800 | .007 | [.00, .07] |
|  | Stimulation × Assessment | *F*(2, 60.66) = 0.12 | .891 | .004 | [.00, .05] |
|  | Measurement × Stimulation × Assessment | *F*(2, 59.96) = 1.08 | .346 | .035 | [.00, .15] |

*Note*. Assessment = t0, t1, t2; Measurement = levels measured immediately before and 30 minutes after consumption of a standardized liquid test meal; Stimulation = verum or sham group.
BDI, Beck's Depression Inventory; BIS-15, Barratt Impulsiveness Scale Short Form; BMI = Body mass index, kg/m², objectively measured; EDE = Eating Disorder Examination; EDE – Q = Eating Disorder Examination- Questionnaire; FCQ-T-r = Food Cravings Questionnaire – Trait; QOLOS, Quality of Life for Obesity Surgery; UPPS, UPPS Impulsive Behavior scale; WHO-5, The World Health Organization-Five Well-Being Index.
^a^ Partial eta squared (η²ₚ) based on Satterthwaite-corrected *F*-values. ^b^ 95% confidence intervals were derived from the noncentral F distribution. Lower bounds of η²ₚ for very small F-values that could not be reliably estimated were reported as 0. Some confidence intervals could not be estimated at all for effects with near-zero *F* values (i.e., Assessment x stimulation interaction for BMI). ^c^ Session was modeled as a continuous predictor (coded 1-6).

## Supplementary Table S4

*Dysfunctional Eating Behaviors as Reported in the EDE*

| Symptom during past 3 months | Measurement point | Verum group (*n =* 9) | Sham group (*n*= 10) | Test statistics |
| --- | --- | --- | --- | --- |
| Plugging: have you had problems with the small opening in your stomach becoming plugged (food getting stuck)?  *N* (%) | t0 | 2 (22%) | 0 | Simple general linear model^1^:  No significant effects were observed for Stimulation (*z* = 0.01, *p* = .996), Session t1 (*z* = –1.17, *p* = .241), Session t2 (*z* = –0.14, *p* = .89), or Stimulation × Session interactions (*z* = –0.005 to 0.000, *p* = .996–1.00). |
|  | t1 | 4 (50%) | 1 (11%) |  |
|  | t2 | 2 (25%) | 0 |  |
| SBEs or “loss of control” eating: eating a subjectively large amount of food with a feeling of a loss of control  *N* (%) | t0 | 2 (22.2%) | 4 (40%) | Mixed-effects logistic regression: No significant effects were observed for Stimulation (*z* = 0.75, *p* = .452), Session t1 (*z* = 0.10, *p* = .924), Session t2 (*z* = 0.10, *p* = .924), or the interaction terms (*z* = –1.19 to –1.18, *p* = .23–.24). |
|  | t1 | 2 (25%) | 1 (11%) |  |
|  | t2 | 2 (25%) | 1 (12.5%) |  |
| Not weight-related vomiting^2^: usually due to plugging, (planned or unplanned), no or little distress  *N* (%) | t0 | 3 (33%) | 3 (30%) | Mixed-effects logistic regression:  No significant effects were observed for Stimulation (*z* = –0.09, *p* = .932), Session t1 (*z* = 1.75, *p* = .081), Session t2 (*z* = 0.86, *p* = .391), or the interaction terms (*z* = –0.40 to 0.84, *p* = .402–.688). |
|  | t1 | 6 (75%) | 6 (66%) |  |
|  | t2 | 4 (50%) | 6 (75%) |  |
| Weight-related vomiting^a^: due to fear of gaining weight or to promote weight loss  *N* (%) | t0 | 0 | 2 (20%) | Mixed-effects logistic regression:  Significant effects were observed for Stimulation (*z* = 6.07, *p* < .001), Session t1 (*z* = 5.50, *p* < .001), Session t2 (*z* = 6.07, *p* < .001), and the interaction terms (*z* = –6.10 to –5.53, *p* < .001). |
|  | t1 | 0 | 1 (11%) |  |
|  | t2 | 1 (12.5%) | 1 (12.5%) |  |
| Picking/nibbling: have you picked at (or nibbled, or grazed) food between meals and snacks. By “picking” I mean eating in an unplanned and repetitious way (excluding loss of control)  *N* (%) | t0 | 3 (33%) | 5 (50%) | Mixed-Effects Logistic Regression:  No significant effects were observed for Stimulation (*z* = 0.71, *p* = .326), Session t1 (*z* = 0.80, *p* = .426), Session t2 (*z* = 0.80, *p* = .426), or the interaction terms (*z* = –0.86 to 0.38, *p* = .392–.704). |
|  | t1 | 4 (50%) | 4 (44%) |  |
|  | t2 | 4 (50%) | 6 (75%) |  |
| Rumination: “In the past four weeks, have you brought food back up to chew it again?”  *N* (%) | t0 | 0 | 0 |  |
|  | t1 | 0 | 0 |  |
|  | t2 | 0 | 0 |  |
| Chewing and spitting out: have you chewed food (put food into your mouth) and spit it out without swallowing it?  *N* (%) | t0 | 0 | 1 (10%) | Simple general linear model^b^:  No significant effects were observed for Stimulation (*z* = 0.01, *p* = .996), Session t1 (*z* = 0.01, *p* = .996), Session t2 (*z* = 0.01, *p* = .996), or the Stimulation × Session interactions (both *z* = 0.01, *p* = .996). |
|  | t1 | 2 (25%) | 2 (22%) |  |
|  | t2 | 2 (25%) | 0 |  |
| Nocturnal eating: an episode of eating after the subject has been to sleep  *N* (%) | t0 | 0 | 1 (10%) | Simple general linear model^b^:  No significant effects were observed for Stimulation (*z* = 0.00, *p* = .998), Session t1 (*z* = 0.00, *p* = 1.00), Session t2 (*z* = 0.00, *p* = 1.00), or the Stimulation × Session interactions (both *z* = 0.00, *p* = 1.00). |
|  | t1 | 0 | 1 (11.1%) |  |
|  | t2 | 0 | 1 (12.5%) |  |

*Note*. EDE = Eating Disorder Examination. ^1^If mixed-effects linear regression did not converge, simple general linear models were applied. ^2^Self-induced and spontaneous vomiting were counted together.
